# Investigating Causality and Shared Genetic Architecture between Neurodegenerative Disorders and Inflammatory Bowel Disease

**DOI:** 10.14336/AD.2022.12209

**Published:** 2023-08-01

**Authors:** Ruijie Zeng, Jinghua Wang, Rui Jiang, Jie Yang, Chunwen Zheng, Huihuan Wu, Zewei Zhuo, Qi Yang, Jingwei Li, Felix W Leung, Weihong Sha, Hao Chen

**Affiliations:** ^1^Department of Gastroenterology, Guangdong Provincial People's Hospital, Guangdong Academy of Medical Sciences, Guangzhou 510080, China.; ^2^The Second School of Clinical Medicine, Southern Medical University, Guangzhou 510515, China.; ^3^Department of Hematology, Guangdong Provincial People's Hospital, Guangdong Academy of Medical Sciences, Guangzhou 510080, China.; ^4^School of Medicine, South China University of Technology, Guangzhou 510006, China.; ^5^Department of Ophthalmology, Guangdong Provincial People's Hospital, Guangdong Academy of Medical Sciences, Guangzhou 510080, China.; ^6^School of Bioscience and Bioengineering, South China University of Technology, Guangzhou 510006, China.; ^7^David Geffen School of Medicine, University of California Los Angeles, Los Angeles, California, USA.; ^8^Sepulveda Ambulatory Care Center, Veterans Affairs Greater Los Angeles Healthcare System, North Hills, California, USA

**Keywords:** Amyotrophic lateral sclerosis, Neurodegenerative disorder, Inflammatory bowel disease, Mendelian randomization, Shared genes

## Abstract

Published observational studies have revealed the connection between neurodegenerative disorders and inflammatory bowel disease (IBD), whereas the causal association remains largely unclear. Our study aims to assess the causality and identify the shared genetic architecture between neurodegenerative disorders and IBD. Two-sample Mendelian randomization analyses were performed to assess the causality between IBD and neurodegenerative disorders (amyotrophic lateral sclerosis [ALS], Alzheimer’s disease [AD], Parkinson’s disease [PD], and multiple sclerosis [MS]). Shared genetic loci, functional interpretation, and transcriptomic profiles were further investigated in ALS and IBD. We identified that genetic predisposition to IBD was suggestively associated with lower odds of ALS (odds ratio [OR] 0.96, 95% confidence interval [CI] 0.94 to 0.99). In contrast, IBD was not genetically associated with an increased risk of AD, PD, or MS (and *vice versa*). Two shared genetic loci (rs6571361 and rs7154847) were derived, and *SCFD1*, *G2E3*, and *HEATR5A* were further identified as novel risk genes with enriched functions related to membrane trafficking. *G2E3* was differentially expressed and significantly correlated with *SCFD1* in patients with ALS or IBD. Our study reveals the suggestively protective role of IBD on ALS, and does not support the causality of AD, PD, or MS on IBD (and *vice versa*). Our findings indicate possible shared genetic architecture and pathways between ALS and IBD. These results provide insights into the pathogenesis and therapeutics of IBD and neurodegenerative disorders.

Inflammatory bowel diseases (IBD), mainly comprised of Crohn’s disease (CD) and ulcerative colitis (UC), are a group of chronic and relapsing-remitting disorders of the intestine with an undefined etiology. Numerous extraintestinal and co-morbid conditions are frequently accompanied by IBD [1]. Neurodegenerative disorders are debilitating diseases characterized by progressive and selective loss of function or structure of neuronal systems [2]. Neurodegenerative disorders include various intractable diseases such as amyotrophic lateral sclerosis (ALS), Alzheimer’s disease (AD), Parkinson’s disease (PD), and multiple sclerosis with no curative therapy [3]. These disorders share an insidious onset and exacerbate irreversibly throughout the disease course, principally occurring in the aging population [4].

The gut-brain axis consists of the bidirectional communications between the intestine and the central nervous system, and such crosstalk indicates the comorbidities of intestinal inflammation and neurological degeneration [5]. Researchers identified that IBD increases the risk of PD in a Danish nationwide cohort involving 7.5 million individuals during a 37-year follow-up [6]. Another study based on the American cohorts demonstrates that a higher incidence of PD is observed among IBD patients [7]. A study based on the Taiwanese National Health Insurance Research Database indicates that IBD is associated with an increased risk of the subsequent development of dementia [8]. The increased prevalence of MS among IBD patients is also verified by a recent meta-analysis [9]. An increasing amount of real-life evidence from epidemiology supports their correlations. In addition, shared mechanisms, for example, autophagy, might be involved in the pathogenesis of IBD and neurodegenerative diseases including ALS [10, 11].

However, the previously reported linkages between IBD and neurodegenerative disorders were mainly observational, while the causality remains largely unexplored. Therefore, it is critical to comprehensively investigate the causal effects of IBD on neurodegenerative disorders using a Mendelian randomization (MR) design.

In this study, the two-sample MR approach was utilized with large-scale genome-wide association study (GWAS) data to evaluate the potential causal relationship between liability to IBD and neurodegenerative disorders, with a main focus on ALS, AD, PD, and MS. Potential genetic links were explored to further elucidate their correlation, and transcriptomic profiles of the shared risk loci were further evaluated.

## MATERIALS AND METHODS

### Data sources

This study relied on publicly available, de-identified and summary-level data mainly from four large-scale cohorts: studies from International Inflammatory Bowel Disease Genetics Consortium (IIBDGC) [12], *Neuron* [13], European Alzheimer & Dementia Biobank (EADB) [14], International Parkinson's Disease Genomics Consortium (IPDGC) [15], and the UK Biobank [16]. The data for IBD (including UC and CD) from the IIBDGC study were based on 25,042 IBD cases, 12,194 CD cases, 12,366 UC cases, and approximately 35,000 control subjects. The ALS GWAS summary data from the study published in *Neuron* contained 20,806 cases diagnosed by EI Escorial criteria and 59,804 controls. The data for AD, which contained 39,106 cases and 401,577 controls, were derived from EADB. The data for PD was based on 33,674 cases and 449,056 control subjects. The data for MS were derived from the UK Biobank and included 456,348 individuals (775 cases and 455,573 control subjects). The descriptions of the studies are provided in Supplementary Table 1.

### Statistical analyses

#### Mendelian randomization analysis

Two-sample MR was performed using the TwoSampleMR package [17]. The instrumental variables were chosen based on the arbitrary *P* value cut-off. A group of single nucleotide polymorphisms (SNPs) with GWAS significance (*P* < 5 × 10^-8^) associated with each trait were selected. The SNPs were clumped by linkage disequilibrium (LD) with an r^2^ < 0.001 and distance (kb) = 5 000 to ensure that the instruments for the exposure were independent. *F* statistics for each instrument were estimated by *F* = β^2^/SE^2^ [18].

For subsequent analysis, inverse variance weighted (IVW) regression was mainly selected for the inference of causality based on three assumptions: 1) variants are associated with the exposure; 2) variants are independent of confounding factors; 3) variants do not directly affect the outcome [19].

The reverse causality was assessed to evaluate whether neurogenerative disorders were causally associated with IBD.

### Sensitivity analysis

For the traits with an IVW *P* < 0.05 and SNP number > 2, heterogeneity tests were performed to evaluate the viability of the effects using Cochran's Q test. Heterogeneity was considered to exist when Cochran’s Q test’s *P* < 0.05 and I^2^ > 50%. MR-Egger and weighted-median (WM) tests were additionally performed to assess the causal effects. MR-Egger regression is based on the assumption that the pleiotropic associates are independent, while it could be inaccurate and largely affected by outlying genetic variants [20]. The WM estimates can provide valid estimates when ≥ 50% of the weight in the analysis comes from the SNVs that are valid instrumental variables [20]. MR test with weighted mode-based estimate (WMBE) was also performed.

Mendelian Randomization Pleiotropy Residual Sum and Outlier (MR-PRESSO) test was performed to identify horizontal pleiotropic outliers [21]. Leave-one-out analysis was used for traits with multiple instruments to assess whether the causality was driven by one single variant.

The causality was accepted following the criteria described in the previous publications: IVW was significant and one of the following assumptions was met: 1) no detected heterogeneity with MR-Egger, WM, and WMBE in the same direction; 2) heterogeneity was detected, while it was corrected by MR-PRESSO (<50% of the instruments were considered outliers); 3) heterogeneity existed with MR-PRESSO test detecting >50% of outliers, while MR Egger and WM were significant with the same direction of effect, and WMBE was in the same direction [21, 22].

We used the *P* value threshold with Bonferroni correction after dividing 0.05 by the number of tests performed. The Bonferroni correction assumes that independent tests were performed. A *P* value < 1.25×10^-2^ (0.05/4, with Bonferroni correction) was considered statistically significant and a *P* value between 1.25×10^-2^ and 0.05 was considered suggestively significant in the MR analyses.

### Genetic correlation analysis and genomic control

Genetic covariance analyzer (GNOVA), which can calculate genetic covariance and estimate genetic correlation according to genetic covariance and heritability, was used to evaluate the genetic correlation between IBD and ALS [23]. The reference data originated from the 1000 Genomes Project European population using default parameters. GNOVA was considered more powerful than conventional cross-trait linkage disequilibrium score regression (LDSC) and adopted as the method for evaluating genetic correlation in our analysis [23, 24]. LDSC was also used to calculate genetic correlations for the selected traits. ANNOVAR was utilized to annotate SNPs in genic and intragenic regions [25]. PLINK-clump was used to prune the SNPs in LD (r^2^ > 0.2 within 250 kb) [26].

### Risk loci identification

Conditional false discovery rate (cFDR) method was used to identify the risk loci showing a strong association with IBD and ALS [27]. cFDRs are characterized as the probability that a certain SNP is falsely positively correlated with the phenotype that the *P* values for both phenotypes (principal and conditional) ≤ the observed *P* values.

$$\mathrm{cFDR}\left(p_{\mathrm{IBD}}|p_{\mathrm{ALS}}\right)=\mathrm{Pr}(H_{0}^{\left(\mathrm{IBD}\right)}|P_{\mathrm{IBD}}\leq p_{\mathrm{IBD}},P_{\mathrm{ALS}}\leq p_{\mathrm{ALS}})$$

As shown above, p_IBD_ indicates the observed significance that a SNP is associated with IBD, and p_ALS_ indicates the observed strength of association that the same SNP is associated with ALS. $H_{0}^{\left(\mathrm{IBD}\right)}$ demonstrates the null hypothesis that a SNP is not correlated with IBD. The SNPs with FDR < 0.01 were considered significant. Conjunctional false discovery rate (conjFDR), which was defined as the maximum of the two cFDR statistics, was further calculated to identify loci that were associated with both IBD and ALS [27].

### Genotyping of the variants and disease risk

The variants were genotyped by the UK Biobank Axiom Array or the UK BiLEVE Axiom Array [28]. The information was coded as 0, 1, and 2 for noncarriers, heterozygous carriers, and homozygous carriers of the minor allele, respectively. We explored the associations between the risk variants and the outcomes (7,400 cases for IBD and 577 cases for ALS) using logistic regression. The risk estimates were adjusted for age, sex, Townsend deprivation index, ethnicity, alcohol consumption, smoking status, metabolic equivalent of task, and body mass index. The results were presented as adjusted odds ratios and 95% confidence intervals (CIs).

### Functional evaluation

The Genotype-Tissue Expression (GTEx) database was used to evaluate the normalized effect size (NES) of the single-tissue cis-expression quantitative trait loci (eQTL) in human tissues [29]. The NES was computed as the effect of the alternative allele compared to the reference allele [29]. The NESs were evaluated in different tissues including adipose, breast, brain, colon, esophagus, heart, lung, muscle, pancreas, skin, spleen, stomach, thyroid, whole blood, artery, nerve, etc. Brain eQTL almanac (Braineac), an online dataset containing data from 10 brain regions obtained from 134 control individuals, was used to investigate eQTL in brain regions, including the cerebellum, frontal cortex, hippocampus, medulla, occipital cortex, putamen, substantia nigra, temporal cortex, thalamus, and white matter [30]. Enrichr (http://amp.pharm.mssm.edu/Enrichr/) was used to assess the shared pathways associated with IBD and ALS, and 3 ontologic terms (biological process, cellular component, and molecular function) were analyzed based on the ontology developed by the Jackson Lab using their MGI-MP browser [31]. The *P* value was computed by Fisher's exact test or the hypergeometric test, and the adjusted *P* value was calculated based on the Benjamini-Hochberg method [31]. The details for the creation of Gene Ontology gene-set libraries for Enrichr are demonstrated in Lists2Networks [32]. The background gene sets include the shared risk genes identified using the conjunctional FDR and eQTL analyses, and genes located within the human leukocyte antigen (HLA) region were excluded because of the complex LD patterns.

### Transcriptomic evaluation of the risk genes

The whole blood expression profiles of ALS patients, IBD patients, and controls were evaluated for the risk genes derived from the above analysis using the datasets GSE112680 and E-MTAB-11349 [33, 34]. The GSE112680 dataset contained a transcriptome-wide analysis of whole blood samples derived from 164 ALS cases and 137 control subjects [33]. The E-MTAB-11349 dataset included 323 blood samples from IBD patients and 267 blood samples from control subjects (data released on March 1, 2022) [34]. The Bioconductor lumi package (v2.44.0) was used for background correction, variance stabilizing transformation, normalization, and quality control of the data [35]. Wilcoxon Rank-Sum test with adjustment by Benjamini-Hochberg method was used to compare the expressions of risk genes between the diseased group and the control group. The ggstatsplot package was used for the evaluation of correlation between gene expression and data visualization [36]. The data were analyzed using R version 4.1.0 (R Project for Statistical Computing, Vienna, Austria) or Python 2.7 (Python Software Foundation, Wilmington, US).

## RESULTS

### Instrumental variable selection

After the clumping process, LD-independent SNPs for IBD were derived, and the following conditions were applied to further exclude the listed SNPs: 1) during the extraction of SNPs from the outcomes (ALS, AD, PD, and MS), a certain requested SNP was not identified and a proxy in LD was not able to be found from the outcome; 2) no correction could be performed for ambiguous SNPs or palindromic SNPs with ambiguous strands. Consequently, the SNPs selected as IV for further analysis would be included in those listed in Supplementary Table 2. *F* statistics for each IV-exposure association were larger than 10 (ranging from 29.86 to 500.60), and therefore the possibility of weak instrumental variable bias was small in our study.

**Table 1:** Mendelian randomization (MR) analysis for the causality of inflammatory bowel diseases (IBD) on neurodegenerative disorders.

				Mendelian randomization				Heterogeneity	
Exposure	Outcome	Method	SNPs	OR	LL	UL	*P*	Q	Q_*P*
**IBD**	ALS	IVW	102	0.96	0.94	0.99	0.03	113.56	0.19
		MR Egger	102	0.98	0.92	1.05	0.66	113.30	0.17
		Weighted median	102	0.97	0.93	1.01	0.13		
		Weighted mode	102	0.98	0.92	1.04	0.41		
		MR PERSSO	102	0.97	0.94	0.99	0.03		
	AD	IVW	79	1.01	0.99	1.02	0.41	54.51	0.98
		MR Egger	79	0.99	0.96	1.03	0.60	53.42	0.98
		Weighted median	79	1.00	0.98	1.03	0.97		
		Weighted mode	79	1.00	0.96	1.03	0.85		
		MR PERSSO	79	1.01	0.99	1.02	0.41		
	PD	IVW	89	1.01	0.98	1.05	0.50	113.84	0.03
		MR Egger	89	1.05	0.97	1.15	0.24	112.59	0.03
		Weighted median	89	1.01	0.95	1.08	0.69		
		Weighted mode	89	1.02	0.95	1.09	0.65		
		MR PERSSO	89	1.01	0.98	1.05	0.50		
	MS	IVW	101	1.04	0.94	1.16	0.43	124.27	0.05
		MR Egger	101	0.80	0.62	1.03	0.08	117.98	0.09
		Weighted median	101	1.01	0.87	1.17	0.94		
		Weighted mode	101	0.99	0.78	1.25	0.91		
		MR PERSSO	101	1.04	0.94	1.16	0.43		

AD: Alzheimer’s disease; ALS: amyotrophic lateral sclerosis; IBD: inflammatory bowel disease; IVW: Inverse variance weighted; LL: lower limits of odds ratio; OR: Odds ratio; PD: Parkinson’s disease; MS: multiple sclerosis; SNP: single-nucleotide polymorphism; UL: upper limits of odds ratio.

**Figure 1.**
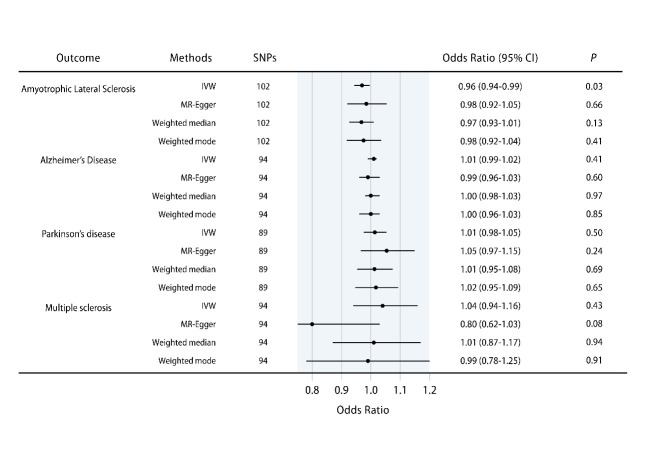
**The causal effects of inflammatory bowel disease (IBD) on amyotrophic lateral sclerosis (ALS), Alzheimer’s disease (AD), Parkinson’s disease (PD), and multiple sclerosis (MS)**. Error bars represent the 95% confidence intervals (CIs) for the estimates. CI: confidence interval; IVW: inverse variance weighted.

### Two-sample Mendelian randomization analysis for causality of IBD on neurodegenerative disorders

**Figure 2.**
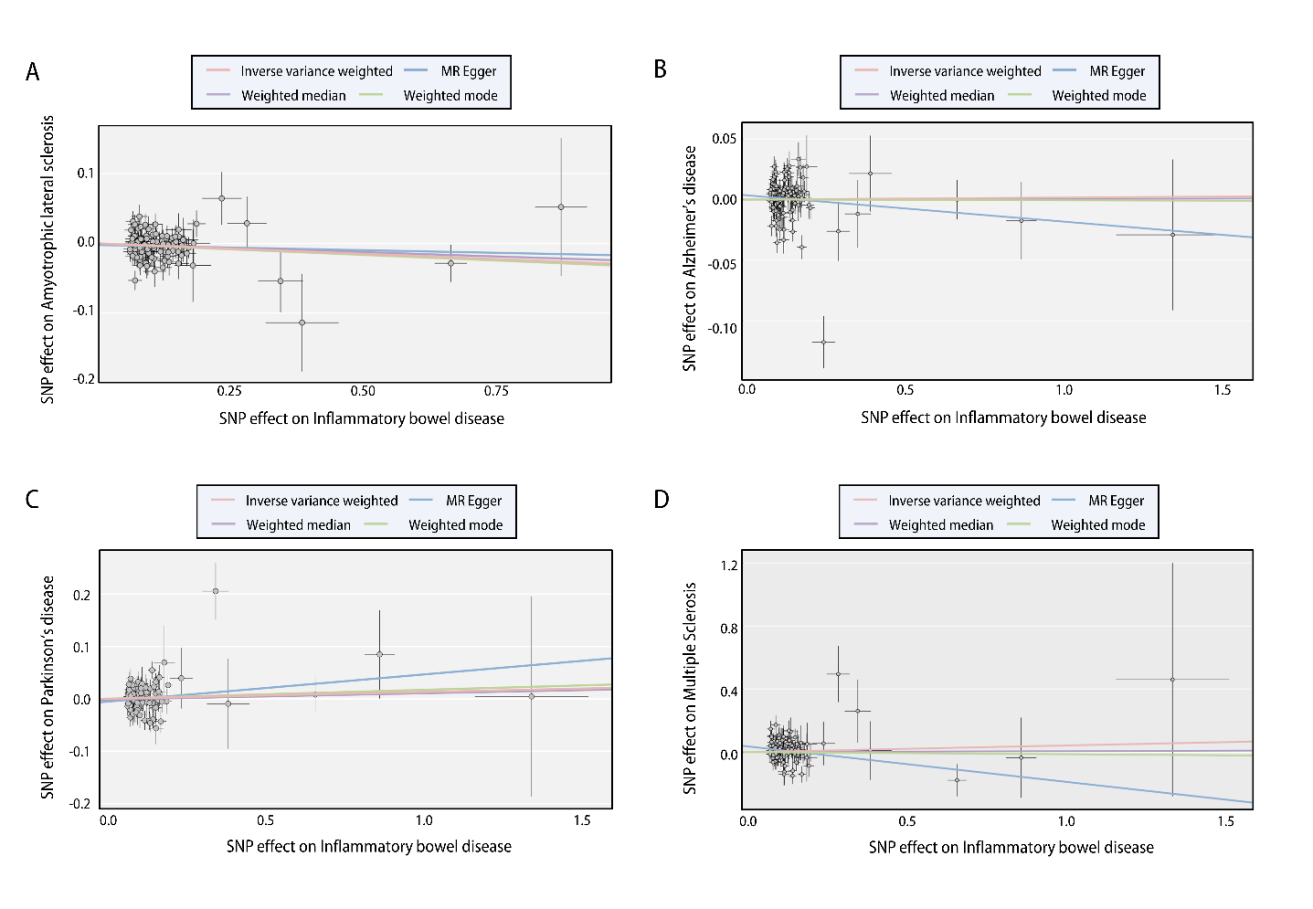
**Scatter plots of the causal effect of inflammatory bowel disease (IBD) on neurodegenerative disorders**. (**A**) IBD on amyotrophic lateral sclerosis (ALS); (B) IBD on Alzheimer’s disease (AD); (C) IBD on Parkinson’s disease (PD); (D) IBD on multiple sclerosis (MS). The slope of each line indicates the estimation of effects by each method. SNP: single nucleotide polymorphism.

The causal effects of IBD on the four major neurodegenerative disorders of interest, including ALS, AD, PD, and MS were explored. Figure 1 and Supplementary Figure 1 summarize the estimates of causal effects of IBD on the four neurodegenerative disorders, and Table 1 demonstrates the detailed information in addition to the MR-PRESSO outlier test and the assessment of heterogeneity. Genetically predicted IBD was suggestively and negatively associated with ALS (IVW [95% confidence interval [CI]]: 0.96 [0.94-0.99], *P* = 0.03; all results from the methods were directionally consistent), and no outliers were detected by MR-PRESSO (Fig. 1, Supplementary Fig. 1A, Table 1). The estimates of causal effect were also demonstrated in scatter plots (Fig. 2A). No evidence of confounding heterogeneity was found by Cochran’s Q test (Q = 113.56, *P* > 0.10; Table 1) and leave-one-out test (Supplementary Fig. 2A). Funnel plots, which provide a visual indication, also demonstrated no heterogeneity for the causal effect of IBD on ALS (Supplementary Fig. 3A). Our results indicated no significant evidence of horizontal pleiotropy for the causality of IBD on ALS (MR-Egger intercept = -0.002, SE = 0.004, *P* = 0.63; MR-PRESSO global test *P* = 0.21; Supplementary Table 4).

In contrast, no causality of IBD on AD (IVW [95% CI]: 1.01 [0.99-1.02], *P* = 0.41; Fig. 1, Supplementary Fig. 1B), PD (IVW [95% CI]: 1.01 [0.98-1.05], *P* = 0.50; Figure 1, Supplementary Fig. 1C) or MS (IVW [95% CI]: 1.04 [0.94-1.16], *P* = 0.43; Fig. 1, Supplementary Fig. 1D) was found. Scatter plots were shown in Fig. 2B-D. Forest plots, leave-one-out test and funnel plots were illustrated in Supplementary Fig. 1B-D, 2B-D and 3B-D, respectively.

### Two-sample Mendelian randomization analysis for causality of CD and UC on neurodegenerative disorders

The two major subtypes of IBD, including CD and UC, were further evaluated for their causal roles in neurodegenerative disorders. Supplementary Table 3 summarizes the estimates of causal effects of CD and UC on the four neurodegenerative disorders. Genetically predicted CD was negatively associated with ALS (IVW [95% CI]: 0.97 [0.95-0.99], *P* = 0.02; all results from the methods were directionally consistent), and no outliers were detected by MR-PRESSO (Supplementary Table 3). No evidence of confounding heterogeneity was found by Cochran’s Q test (IVW Q = 77.55, *P* > 0.10; Supplementary Table 3). Our results indicated no significant evidence of horizontal pleiotropy for the causality of CD on ALS (MR-Egger intercept = -0.008, SE = 0.005, *P* = 0.12; MR-PRESSO global test *P* = 0.46; Supplementary Table 4). In addition, the causality of UC on MS was also suggestively significant (IVW [95% CI]: 0.88 [0.78-0.99], *P* = 0.03; Supplementary Table 3)

In contrast, no causality of UC on ALS, or CD on MS was identified. No causal relationships were found for CD or UC on AD or PD. The risk estimates were demonstrated in Supplementary Table 3.

### Two-sample Mendelian randomization analysis for causality of neurodegenerative disorders on IBD

The causal effects of ALS (IVW [95% CI]: 1.00 [0.89-1.12], *P* > 0.05), AD (IVW [95% CI]: 0.99 [0.94-1.05], *P* > 0.05), PD (IVW [95% CI]: 1.03 [0.99-1.08], *P* > 0.05), or MS (Wald ratio [95% CI]: 0.94 [0.86-1.03], *P* > 0.05) on IBD were not significant (Supplementary Table 5). No causal relationships were found for the above neurodegenerative disorders on UC or CD (Supplementary Table 5).

**Table 2:** Genetic correlation between inflammatory bowel disease and neurodegenerative disorders.

Genetic Covariance Analyzer			
Disease	ρ (SE)	*P*	Genetic Correlation
**IBD-ALS**	-0.0472 (0.0057)	**<0.0001**	-0.3255
**IBD-AD**	-0.0031 (0.0029)	0.2786	-0.0279
**IBD-PD**	0.0019 (0.0022)	0.4023	-0.0242
**IBD-MS**	-0.0011 (0.0022)	0.6103	-0.0346
**Linkage Disequilibrium Score Regression**			
**Disease**	**Genetic Correlation (SE)**	***P***	**Z-score**
**IBD-ALS**	-0.1576 (0.0772)	**0.0412**	-2.0416
**IBD-AD**	0.0147 (0.0461)	0.7495	0.3193
**IBD-PD**	0.0138 (0.0404)	0.7321	0.3424
**IBD-MS**	-0.1624 (0.2798)	0.5616	-0.5804

AD: Alzheimer’s disease; ALS: amyotrophic lateral sclerosis; IBD: inflammatory bowel disease; PD: Parkinson’s disease; SE: standard error.

**Figure 3.**
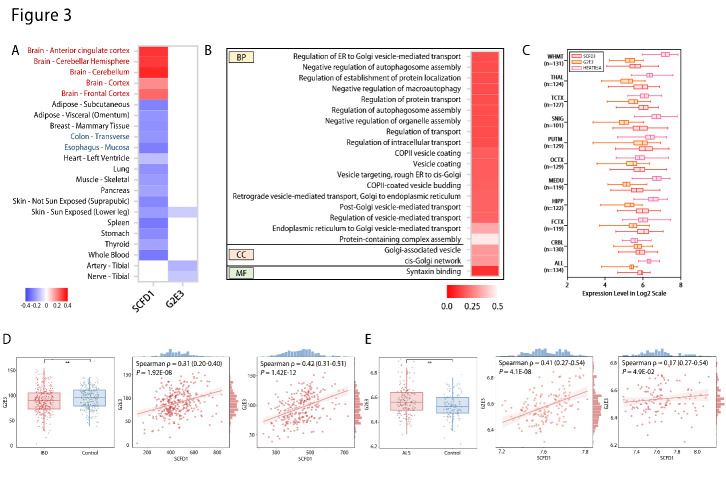
**Functional interpretation and transcriptomic analysis of shared risk loci**. (**A**) Expression quantitative trait loci (eQTL) of shared genetic loci and normalized effect size (NES) of eQTLs in different tissues by GTEx analysis. Blue: negative NES. Red: positive NES. (**B**) Enriched pathways related to eQTLs. Deeper color indicates a more significant *P* value. (**C**) Expressions of *SCFD1, G2E3,* and *HEATR5A* in different brain regions by Braineac database. (**D**) mRNA expressions of *G2E3* in patients with inflammatory bowel disease (IBD) and control subjects (left panel). Correlations between *SCFD1* expression and *G2E3* expression in IBD patients (middle panel) and control subjects (right panel) from GSE112680. (**E**) mRNA expressions of *G2E3* in patients with inflammatory bowel disease (IBD) and control subjects (left panel). Correlations between *SCFD1* expression and *G2E3* expression in ALS patients (middle panel) and control subjects (right panel) from E-MTAB-11349. ALL, average of all regions; ALS: amyotrophic lateral sclerosis; COPII: coat protein complex II; CRBL, cerebellar cortex; ER: endoplasmic reticulum; FCTX, frontal cortex; HIPP, hippocampus; IBD: inflammatory bowel disease; MEDU, medulla; OCTX, occipital cortex; PUTM, putamen; SNIG, substantia nigra; TCTX, temporal cortex; THAL, thalamus; WHMT, intralobular white matter. * *P* < 0.05, ** *P* < 0.01, *** *P* < 0.001.

### Genetic correlation between IBD and ALS and identification of shared risk loci

A significantly negative genetic correlation between IBD and ALS was identified (genetic correlation = -0.326, *P* < 1.00×10^-3^ by GNOVA; genetic correlation = -0.158, *P* = 4.10×10^-2^ by LDSC; Table 2). Conjunctional FDR analysis was further performed to explore genetic variants associated with IBD conditional on ALS. Two risk loci were further identified, namely rs6571361 (*P* = 2.54×10^-7^, FDR = 3.48×10^-2^; *SCFD1*) and rs7154847 (*P* = 4.46×10^-7^, FDR = 2.70×10^-2^; *G2E3*) (Supplementary Table 6). Compared with variant noncarriers, the homozygous variant carriers had higher risks of developing ALS (adjusted OR 1.42 [1.16-1.69], *P* = 0.03 for rs6571361; adjusted OR 1.48 [1.09-1.77], *P* = 0.01 for rs7154847; Supplementary Table 7).

### Exploration of potential function of shared risk loci

To interpret the function of shared risk loci identified by the conjunction FDR method, cis- eQTL was evaluated in GTEx database. The pleiotropic risk loci affect Sec1 Family Domain Containing 1 (SCFD1) and G2/M phase-specific E3 ubiquitin-protein ligase (G2E3) in tissues from both GTEx and Brain eQTL almanac (Braineac) (Fig. 3A, Table 3). In addition, the pleiotropic risk loci can affect HEAT repeat-containing protein 5A (HEATR5A) in brain tissues (Table 3). Based on GTEx analysis, the NES of *SCFD1* was generally higher in brain tissues, whereas that of *SCFD1* was significantly lower in the digestive tract, including esophageal mucosa and colon (Fig. 3A). The NES of G2E3 was only significantly lower in three tissues, including non-sun-exposed skin, tibial artery, and tibial nerve (Fig. 3A). Pathway enrichment analysis was further performed to identify biological pathways identified by cis-eQTL analysis. Twenty pathways of biological processes were significantly enriched, mainly including vesicle-mediated transport and autophagy-related pathways (*P* < 0.05, Fig. 3B, Supplementary Table 8). For pathways of cellular components, the Golgi-associated pathways were significantly enriched (*P* < 0.01, Fig. 3B, Supplementary Table 8). Syntaxin binding pathway was significantly enriched for the pathway of molecular function (*P* < 0.01, Fig. 3B, Supplementary Table 8). Furthermore, the expressions of *SCFD1*, *G2E3*, and *HEATR5A* in brain regions were evaluated using the Braineac database. The expression of *SCFD1* was highest in putamen, while that of *SCFD1* was lowest in white matter (Log2 fold change between expression in putamen/white matter = 1.4, *P* = 3.0×10^-12^; Fig. 3C). *G2E3* expression was highest in putamen, whereas *G2E3* expression was lowest in substantia nigra (Log2 fold change between expression in putamen/substantia nigra = 1.9, *P* = 5.9 × 10^-24^; Fig. 3C). The expression of *HEATR5A* was highest in white matter. In contrast, the expression of *HEATR5A* was lowest in cerebellar cortex (Log2 fold change between expression in putamen/white matter = 3.0, *P* = 7.8 × 10^-74^; Fig. 3C).

**Table 3:** Expression quantitative trait loci (eQTL) indicating the functional effects of shared risk single nucleotide polymorphisms (SNPs) in human brain tissue.

Gene Symbol	Position	Chr	start	stop	*P*
***G2E3***	31199109	chr14	31028362	31089250	**<0.01**
***G2E3***	31183168	chr14	31028362	31089250	**0.01**
***HEATR5A***	31183168	chr14	31760322	31889973	**0.01**
***SCFD1***	31183168	chr14	31091515	31223811	**0.02**
***HEATR5A***	31183168	chr14	31760322	31889973	**0.02**
***HEATR5A***	31183168	chr14	31760322	31889973	**0.03**
***HEATR5A***	31183168	chr14	31760322	31889973	**0.04**
***HEATR5A***	31199109	chr14	31760322	31889973	**0.04**

Chr: chromosome; SNP: Single nucleotide polymorphism.

### Differential expression of the risk genes and correlation of gene expressions

The mRNA expressions of *G2E3, SCFD1,* and *HEATR5A* in blood samples of patients with IBD or ALS were further evaluated. The expressions of *G2E3* in blood samples of patients with IBD were significantly lower than that of the control (*P* = 0.0036; Fig. 3D), and positive correlations between *G2E3* expression and *SCFD1* expression were observed in both patients with IBD (Spearman ρ [95% CI] = 0.31 [0.20-0.40], *P* = 1.92×10^-8^_;_ Fig. 3D) and control subjects (Spearman ρ [95% CI] = 0.42 [0.31-0.51], *P* = 1.42×10^-12^; Fig. 3D). In contrast, *G2E3* expressions in ALS patients were significantly higher than those of the control group (*P* = 0.0038; Fig. 3E). Positive correlations between the expression of *G2E3* and *SCFD1* were identified in both the ALS diseased group (Spearman ρ [95% CI] = 0.41 [0.27-0.54], *P* = 4.1×10^-8^_;_ Fig. 3E) and the control group (Spearman ρ [95% CI] = 0.41 [0.27-0.54], *P* = 4.9×10^-2^_;_ Fig. 3E). The expressions of *SCFD1* or *HEATR5A* were not significantly altered, or slightly altered in blood samples of patients with IBD or ALS (Supplementary Figure 4A-D). The correlations between expressions of *G2E3* and *HEATR5A, or SCFD1* and *HEATR5A*, were not identified in patients with IBD or ALS, or controls (Supplementary Fig. 4E-L).

## DISCUSSION

By leveraging large GWAS datasets, our study indicates that the causality of AD, PD or MS on IBD (and *vice versa*) is unfounded, whereas the genetic liability to IBD is suggestively protective for ALS. Our findings provide reassurance for patients suffering from neurodegenerative disorders and IBD.

ALS is a rapidly progressive neuromuscular disease characterized by dysfunction of both upper and lower motor neurons, and most ALS patients die within 3 to 5 years due to respiratory failure [37]. The pathogenesis of ALS is multifactorial and involves complex interactions among diverse environmental and genetic factors. Defects in vesicular trafficking and altered neuronal functions are indicated as one of the pathogenic mechanisms in ALS. For IBD, altered morphology of vesicles in colonic mucosa cells increases susceptibility to experimental colitis, and autophagy is regarded as a central issue in IBD development [10, 38]. The shared risk genes could have functional relevance to both IBD and ALS. *SCFD1* plays important roles in mediating vesicle transport and membrane-fusion events, as well as autophagy as indicated by the pathway enrichment results [39]. Recent studies have also identified that *SCFD1* is one of the most significant genes that mediate the risk of ALS [40]. Our findings indicate that the expression of *G2E3* is reduced in patients with IBD, whereas that of *G2E3* is increased in patients with ALS, and the expression of *G2E3* in the two diseases was positively correlated with the expression of *SCFD1*, which could simultaneously exert its functions. Therefore, the decreased expression in patients with IBD might serve as a protective factor for neurodegeneration, contributing to the casual association of IBD with a decreased risk of ALS. Currently, little is known about *G2E3*, and its functions in inflammatory bowel disease or neurodegenerative disorders remain to be explored.

Two recent studies tried to evaluate the causality between IBD and neurodegenerative disorders [41, 42]. However, in-depth evaluation with shared genes was not performed [41, 42]. Intriguingly, although previously published research demonstrated potential causal effect of MS on IBD [43], our study does not support the causality. Our study also does not support a causal relationship between IBD and AD or PD, which is contradictory to previous observational studies [6-9]. Because a high prevalence of anxiety and depression is observed in patients with IBD, and unsubstantiated concerns would tremendously aggravate their psychological comorbidities, which in turn worsen disease outcomes, unnecessary worries should be avoided for patients [44].

The major strength of our study is the two-sample MR design, which limits the confounding and reverse-causality bias in observational studies, with the use of strong instruments. Our results are robust and with no significant evidence of horizontal pleiotropy. In addition, we identified novel SNPs associated with ALS and IBD, which provide plausible explanations for their correlation and deepen current understandings of the disorders. The shared risk genes were validated by transcriptomic analysis. Our study has several limitations. Our study merely involves the European population, which limits the universality of our findings to other ancestries. Due to the lack of publicly available GWAS summary data or rarity in nature, the causality of IBD on other neurodegenerative disorders was not assessed. Moreover, although the significance of the causality for IBD on ALS is only suggestive, Bonferroni correction can be considered overly conservative [45], and further investigations with larger and more powerful datasets are warranted.

In summary, the causality of AD, PD, or MS on IBD (and *vice versa*) is not supported by current evidence, whereas IBD is suggestively protective to ALS. We suggest the possible genetic architecture shared between ALS and IBD and indicate that modulation of membrane trafficking-related pathways and dysregulation of *G2E3* might contribute to their pathogenesis.

## Supplementary Materials

The Supplementary data can be found online at: www.aginganddisease.org/EN/10.14336/AD.2022.1209. GWAS summary statistics are available from the original manuscript of each study in Supplementary Table 1 and GWAS Catalog (https://www.ebi.ac.uk/gwas/). Code used in this study is available from the corresponding authors upon reasonable request.
