# Supplementary material for: Investigating Causality and Shared Genetic Architecture between Neurodegenerative Disorders and Inflammatory Bowel Disease
Source: Aging Dis. 2023 Aug 1;14(4):1349–59. doi: 10.14336/AD.2022.12209 (PMC10389839; doi:10.14336/AD.2022.12209)
Supplement: Supplementary file 1 — The Supplementary data can be found online at: www.aginganddisease.org/EN/10.14336/AD.2022.1209. GWAS summary statistics are available from the original manuscript of each study in Supplementary Table 1 and GWAS Catalog (https://www.ebi.ac.uk/gwas/). Code used in this study is available from the corresponding authors upon reasonable request. [file AD-14-4-1349-s.pdf]

SUPPLEMENTARY DATA

Investigating Causality and Shared Genetic Architecture between Neurodegenerative Disorders and Inflammatory Bowel Disease

Ruijie Zeng, Jinghua Wang, Rui Jiang, Jie Yang, Chunwen Zheng, Huihuan Wu, Zewei Zhuo, Qi Yang, Jingwei Li, Felix W. Leung, Weihong Sha, Hao Chen

© 2022. Zeng R et al. Published online at http://www.aginganddisease.org/EN/10.14336/AD.2022.1209

SUPPLEMENTARY DATA

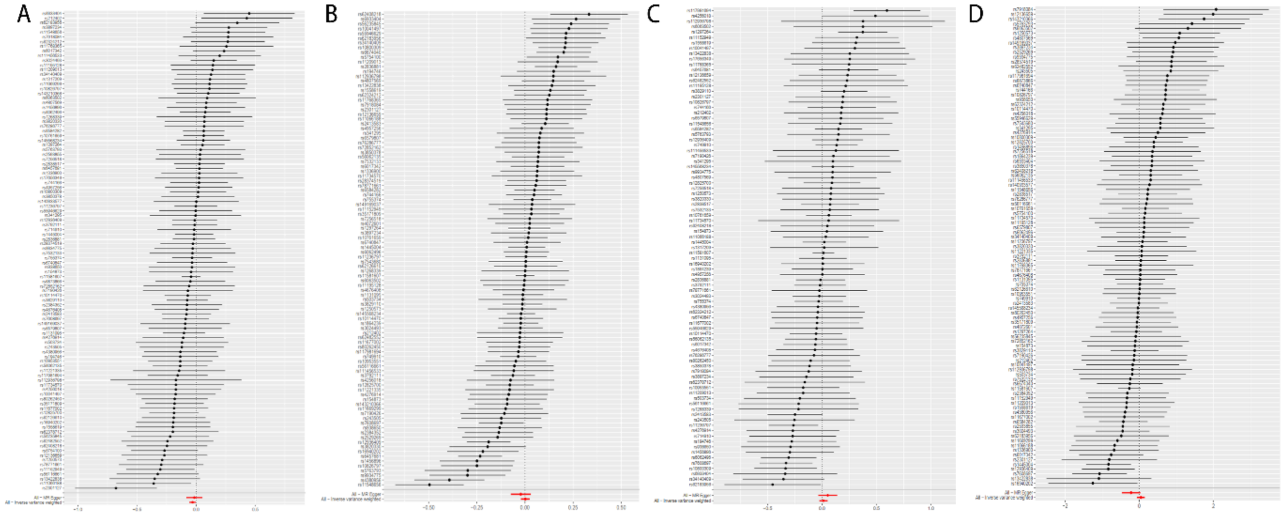

Supplementary Figure 1. Forest plots illustrate the effect of inflammatory bowel disease (IBD) on neurodegenerative disorders. **A**. IBD on amyotrophic lateral sclerosis (ALS); **B**. IBD on Alzheimer’s disease (AD); **C**. IBD on Parkinson’s disease (PD); **D.** IBD on multiple sclerosis (MS).

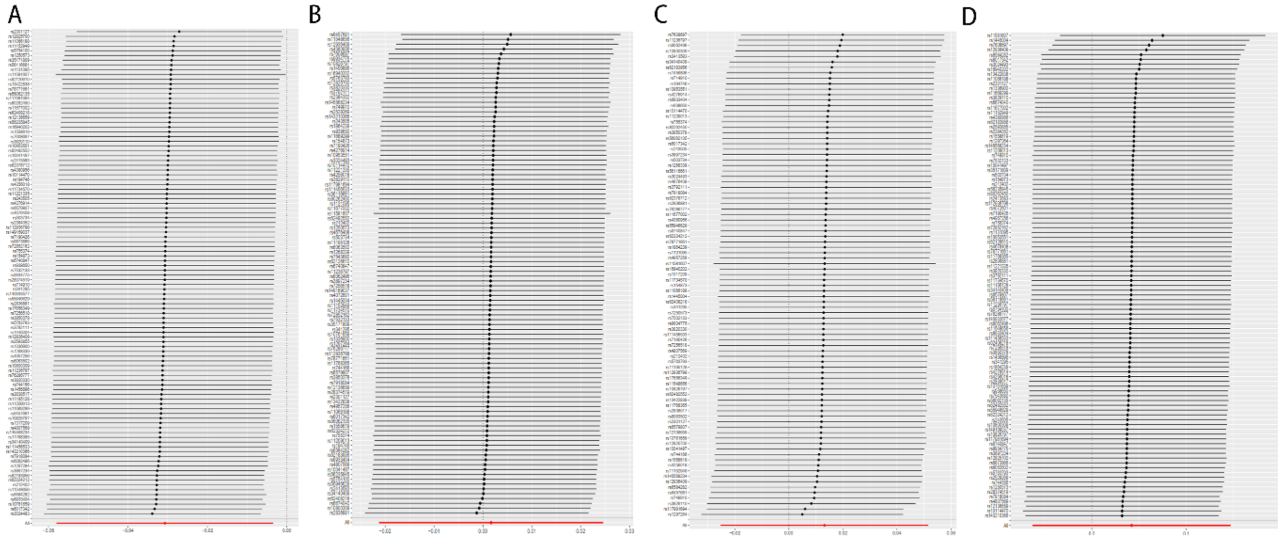

Supplementary Figure 2. Leave-one-out sensitivity analysis for inflammatory bowel disease (IBD) on neurodegenerative disorders. A. IBD on amyotrophic lateral sclerosis (ALS); **B.** IBD on Alzheimer’s disease (AD); **C.** IBD on Parkinson’s disease (PD); **D.** IBD on multiple sclerosis (MS).

© 2022. Zeng R et al. Published online at http://www.aginganddisease.org/EN/10.14336/AD.2022.1209

SUPPLEMENTARY DATA

Supplementary Figure S3

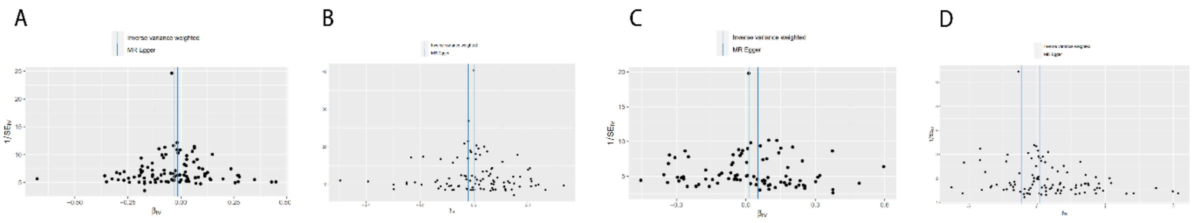

Supplementary Figure S3. Funnel plots evaluating the horizontal heterogeneity for the effect of inflammatory bowel disease (IBD) on neurodegenerative disorders. A. IBD on amyotrophic lateral sclerosis (ALS); **B.** IBD on Alzheimer's disease (AD); **C.** IBD on Parkinson's disease (PD); **D.** IBD on multiple sclerosis (MS).

Supplementary Figure S4

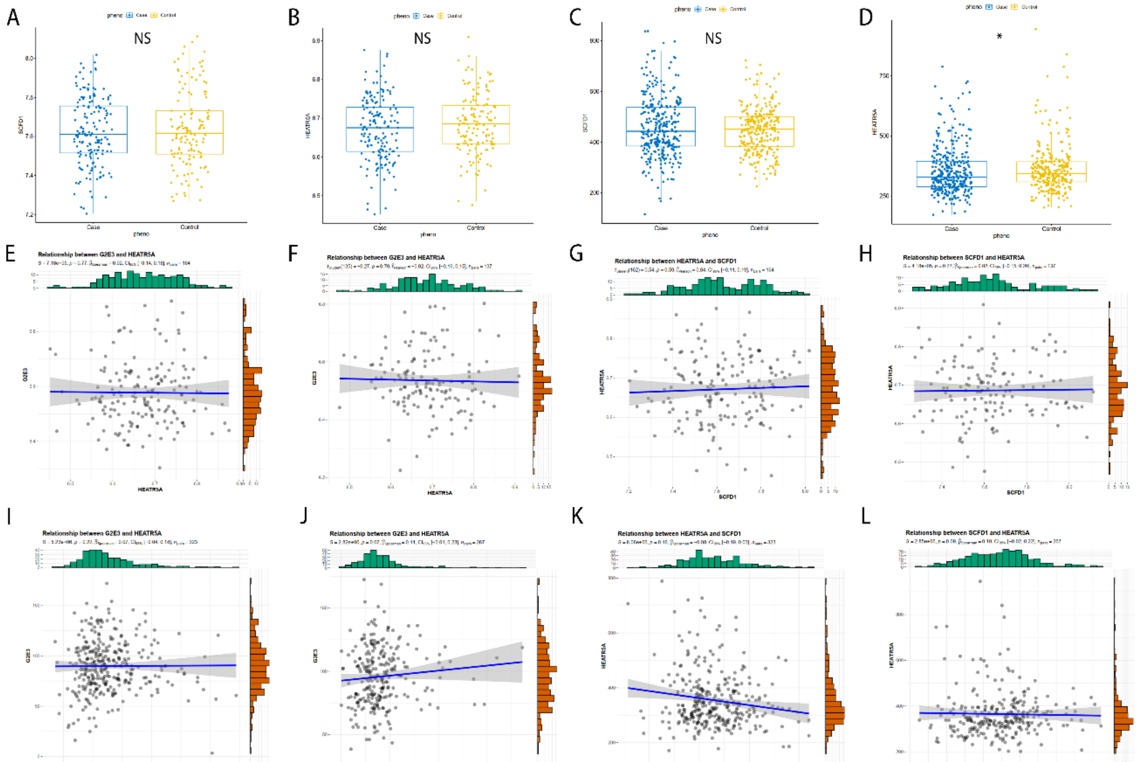

Supplementary Figure S4. Transcriptomic analysis of shared risk loci. A-B. mRNA expressions of SCFD1 **(A)** and HEATR5A **(B)** in IBD patients and control subjects from GSE112680. **C-D.** mRNA expressions of SCFD1 **(C)** and HEATR5A **(D)** in ALS patients and control subjects from E-MTAB-11349. **E-F.** Correlations between *G2E3* expression and *HEATR5A* expression in IBD patients **(E)** and control subjects **(F)** from GSE112680. **G-H.** Correlations between *SCFD1* expression and *HEATR5A* expression in IBD patients **(G)** and control subjects **(H)** from GSE112680. **I-J.** Correlations between *G2E3* expression and *HEATR5A* expression in ALS patients **(I)** and control subjects **(J)** from E-MTAB-11349. **K-L.** Correlations between *SCFD1* expression and *HEATR5A* expression in ALS patients **(K)** and control subjects **(L)** from E-MTAB-11349. * $P < 0.05$, ** $P < 0.01$, *** $P < 0.001$, NS: non-significant.

© 2022. Zeng R et al. Published online at http://www.aginganddisease.org/EN/10.14336/AD.2022.1209

SUPPLEMENTARY DATA

Supplementary Table 1. Characteristics of data sources used in this study

Traits	Sample size (cases/controls)	Ancestry	Reference
IBD	25,042/34,915	European	de Lange et al., 2017
CD	12,194/28,072		
UC	12,366/33,609		
ALS	20,806/59,804	European	Nicolas et al., 2018
AD	39,106/401,577	European	Bellenguez et al., 2022
PD	33,674/449,056	European	Nalls et al., 2019
MS	775/455,573	European	Jiang et al., 2021

AD: Alzheimer’s disease; ALS: amyotrophic lateral sclerosis; CD: Crohn’s disease; IBD: inflammatory bowel disease; MS: multiple sclerosis; PD: Parkinson’s disease; UC: ulcerative colitis.

References

Bellenguez, C., Küçükali, F., Jansen, I.E., Kleineidam, L., Moreno-Grau, S., Amin, N., Naj, A.C., Campos-Martin, R., Grenier-Boley, B., and Andrade, V. (2022). New insights into the genetic etiology of Alzheimer’s disease and related dementias. ***Nat Genet*** 54, 412-436.

de Lange, K.M., Moutsianas, L., Lee, J.C., Lamb, C.A., Luo, Y., Kennedy, N.A., Jostins, L., Rice, D.L., Gutierrez-Achury, J., Ji, S.G., et al. (2017). Genome-wide association study implicates immune activation of multiple integrin genes in inflammatory bowel disease. ***Nat Genet*** 49, 256-261.

Jiang, L., Zheng, Z., Fang, H., and Yang, J. (2021). A generalized linear mixed model association tool for biobank-scale data. ***Nat Genet*** 53, 1616-1621.

Nalls, M.A., Blauwendraat, C., Vallerga, C.L., Heilbron, K., Bandres-Ciga, S., Chang, D., Tan, M., Kia, D.A., Noyce, A.J., Xue, A., et al. (2019). Identification of novel risk loci, causal insights, and heritable risk for Parkinson's disease: a meta-analysis of genome-wide association studies. ***Lancet Neurol*** 18, 1091-1102.

Nicolas, A., Kenna, K.P., Renton, A.E., Ticozzi, N., Faghri, F., Chia, R., Dominov, J.A., Kenna, B.J., Nalls, M.A., Keagle, P., et al. (2018). Genome-wide Analyses Identify KIF5A as a Novel ALS Gene. ***Neuron*** 97, 1268-1283.e1266.

© 2022. Zeng R et al. Published online at http://www.aginganddisease.org/EN/10.14336/AD.2022.1209

SUPPLEMENTARY DATA

Supplementary Table 2. Detailed information for SNPs in the Mendelian randomization analysis of inflammatory bowel disease (IBD) on neurodegenerative disorders.

SNP	Chr	Location	Association with ALS			Association with AD			Association with PD			Association with MS		
			β	SE	*P*	β	SE	*P*	β	SE	*P*	β	SE	*P*
rs10041497	5	142135017	-0.17	0.17	0.31	/	/	/	0.30	0.24	0.20	-0.18	0.64	0.78
rs10114470	9	114785492	-0.07	0.1	0.48	-0.02	0.06	0.71	-0.05	0.12	0.66	0.64	0.37	0.09
rs10761659	10	62685804	0.06	0.09	0.46	0.02	0.05	0.74	0.06	0.11	0.56	0.16	0.32	0.61
rs10800309	1	161502368	0.02	0.12	0.89	/	/	/	-0.33	0.15	0.02	0.44	0.44	0.32
rs10826797	10	30401447	0.11	0.15	0.47	/	/	/	0.19	0.25	0.46	0.70	0.56	0.21
rs10953551	7	107840456	-0.13	0.14	0.33	-0.05	0.08	0.55	-0.17	0.17	0.31	<0.01	0.50	1.00
rs11066188	12	112172910	-0.36	0.16	0.03	0.11	0.09	0.24	0.03	0.20	0.86	-0.67	0.59	0.26
rs111456533	10	124750812	0.2	0.18	0.26	-0.06	0.11	0.58	0.13	0.29	0.67	0.30	0.67	0.65
rs11152949	6	106001210	-0.3	0.15	0.04	0.03	0.09	0.70	0.32	0.20	0.10	-0.33	0.54	0.53
rs11195128	10	110426390	0.14	0.18	0.44	-0.01	0.11	0.96	0.22	0.29	0.45	0.13	0.68	0.85
rs11209013	1	67190603	0.14	0.18	0.44	0.17	0.11	0.11	-0.17	0.22	0.43	-0.34	0.66	0.60
rs11221335	11	128516011	-0.14	0.2	0.47	-0.08	0.12	0.50	/	/	/	0.06	0.75	0.93
rs11236797	11	76588605	<0.01	0.09	0.98	0.01	0.06	0.92	-0.26	0.12	0.02	0.08	0.34	0.81
rs112936798	1	39336709	-0.17	0.28	0.54	0.15	0.14	0.29	0.38	0.38	0.33	-0.19	0.82	0.82
rs1131095	3	49676792	-0.1	0.09	0.25	-0.01	0.05	0.84	0.01	0.11	0.95	0.02	0.34	0.95
rs11548656	16	81883307	0.27	0.16	0.09	/	/	/	0.17	0.24	0.49	0.23	0.58	0.69
rs11581607	1	67242007	-0.04	0.04	0.29	0.00	0.02	1.00	0.01	0.05	0.80	-0.27	0.16	0.08
rs11669299	19	10385945	0.11	0.16	0.49	-0.10	0.10	0.31	/	/	/	-0.59	0.58	0.31
rs11677002	2	28391534	-0.19	0.15	0.2	-0.03	0.09	0.75	-0.05	0.20	0.82	-0.40	0.55	0.46
rs11734570	4	38586832	-0.17	0.21	0.41	0.06	0.12	0.60	0.06	0.33	0.85	0.13	0.76	0.86
rs11768365	7	6505557	0.26	0.2	0.21	0.12	0.12	0.32	0.25	0.27	0.36	0.04	0.73	0.96
rs117981694	12	40428296	-0.16	0.13	0.23	-0.03	0.08	0.67	0.60	0.16	<0.01	0.76	0.58	0.19
rs12136659	1	172875110	-0.27	0.19	0.15	0.11	0.11	0.30	0.23	0.23	0.31	1.99	0.68	<0.01
rs1250573	10	79282718	-0.29	0.15	0.06	-0.01	0.09	0.88	0.08	0.25	0.75	1.09	0.55	0.05
rs1268339	1	1280044	0.07	0.21	0.73	<0.01	0.12	0.99	-0.22	0.28	0.44	/	/	/
rs12825700	12	68099200	-0.19	0.11	0.08	-0.08	0.06	0.22	0.09	0.13	0.50	0.39	0.40	0.32
rs12936409	17	39887396	-0.01	0.1	0.9	/	/	/	0.14	0.12	0.23	-0.84	0.36	0.02
rs1297264	21	15443698	0.05	0.09	0.57	0.02	0.06	0.69	0.37	0.12	<0.01	-0.08	0.35	0.82
rs1317209	1	19813543	0.12	0.15	0.44	/	/	/	0.02	0.25	0.94	/	/	/
rs1336900	1	150706557	0.03	0.16	0.87	0.06	0.10	0.52	0.25	0.28	0.36	-0.68	0.62	0.27
rs13422838	2	186638119	-0.35	0.2	0.07	0.14	0.12	0.23	/	/	/	-1.09	0.72	0.13
rs140933577	13	40262133	<0.01	0.18	1	/	/	/	/	/	/	0.27	0.72	0.70
rs143210366	6	31524576	0.1	0.13	0.45	-0.09	0.09	0.28	/	/	/	1.74	0.63	0.01
rs1445004	5	40414317	-0.02	0.08	0.83	0.01	0.05	0.86	0.02	0.10	0.84	-0.83	0.31	0.01
rs145568234	6	32279268	0.06	0.12	0.6	-0.02	0.04	0.58	0.10	0.10	0.31	-0.04	0.29	0.89
rs1456896	7	50264865	0.08	0.17	0.62	/	/	/	-0.30	0.21	0.15	0.39	0.61	0.52
rs149169037	7	20537675	-0.09	0.19	0.63	0.04	0.11	0.75	/	/	/	0.97	0.67	0.15
rs154873	20	59279977	-0.04	0.18	0.81	-0.09	0.10	0.38	0.04	0.26	0.87	-0.12	0.65	0.85
rs1558619	2	102315090	-0.2	0.16	0.22	0.13	0.10	0.17	0.31	0.21	0.13	-0.35	0.61	0.56
rs16940202	16	85980635	-0.19	0.16	0.22	/	/	/	0.01	0.19	0.97	-1.25	0.61	0.04
rs17656349	5	150226431	0.02	0.19	0.9	/	/	/	0.25	0.31	0.41	/	/	/
rs1864239	15	79913012	/	/	/	-0.02	0.05	0.64	<0.01	0.14	0.98	0.34	0.55	0.53
rs194746	14	68816170	-0.13	0.16	0.41	0.15	0.10	0.13	-0.28	0.21	0.18	/	/	/
rs212402	6	159051263	0.43	0.2	0.03	-0.03	0.11	0.82	0.17	0.27	0.51	-0.15	0.72	0.84
rs2301127	16	11273620	-0.68	0.18	<0.01	0.11	0.10	0.28	0.19	0.22	0.39	-0.81	0.66	0.22
rs2384352	16	11273620	-0.08	0.15	0.61	-0.14	0.09	0.13	-0.25	0.13	0.05	-0.32	0.57	0.57
rs2413583	22	39263768	-0.08	0.11	0.44	0.11	0.06	0.09	-0.25	0.28	0.37	-0.01	0.40	0.98
rs243505	17	148738247	-0.12	0.18	0.5	-0.12	0.11	0.24	/	/	/	0.85	0.65	0.19
rs2529269	7	74869879	/	/	/	-0.14	0.09	0.13				0.91	0.56	0.10
rs2593855	3	71126344	0.03	0.18	0.85	/	/	/	/	/	/	-0.45	0.64	0.49
rs2836881	21	39094373	-0.02	0.09	0.84	/	/	/	-0.01	0.12	0.90	0.04	0.35	0.91
rs28374519	16	28478021	-0.03	0.14	0.85	0.06	0.08	0.44	/	/	/	0.86	0.49	0.08
rs2838517	21	44193942	0.03	0.11	0.8	/	/	/	0.07	0.14	0.59	0.22	0.41	0.58
rs3024493	1	206770623	0.15	0.1	0.14	-0.02	0.06	0.69	-0.03	0.13	0.84	-0.45	0.37	0.22
rs341295	5	112513193	-0.01	0.2	0.98	0.08	0.12	0.49	0.10	0.32	0.75	0.57	0.74	0.44
rs34140409	6	31955877	0.13	0.15	0.39	/	/	/	/	/	/	0.11	0.58	0.85
rs35171809	6	167019278	-0.18	0.13	0.15	0.03	0.07	0.66	-0.35	0.19	0.06	-0.06	0.47	0.89
rs3792111	2	233271044	-0.01	0.1	0.89	-0.06	0.06	0.28	-0.02	0.12	0.88	0.06	0.37	0.87
rs3820330	1	19815920	0.07	0.17	0.68	-0.19	0.10	0.05	0.08	0.23	0.73	0.07	0.62	0.91
rs3829110	9	136374746	-0.08	0.09	0.39	-0.01	0.05	0.80	0.20	0.11	0.06	-0.13	0.33	0.70
rs3850378	14	87951173	0.01	0.16	0.92	0.07	0.09	0.43	-0.12	0.19	0.55	0.33	0.57	0.56
rs3897234	13	26967893	0.28	0.17	0.1	0.02	0.10	0.84	-0.14	0.22	0.51	0.92	0.63	0.14
rs4072601	17	72645188	/	/	/	0.02	0.09	0.79	/	/	/	-0.07	0.57	0.91
rs4256018	20	6113242	-0.17	0.2	0.38	-0.08	0.12	0.52	0.49	0.25	0.05	0.62	0.73	0.39

© 2022. Zeng R et al. Published online at http://www.aginganddisease.org/EN/10.14336/AD.2022.1209

rs4276914	1	155169753	-0.11	0.17	0.53	-0.08	0.10	0.42	-0.27	0.22	0.22	0.50	0.65	0.44
rs4380956	8	125516832	-0.13	0.16	0.39	/	/	/	-0.04	0.19	0.85	-0.38	0.58	0.51
rs4676408	2	240634984	-0.08	0.14	0.57	-0.01	0.08	0.92	-0.07	0.18	0.72	0.02	0.51	0.96
rs4807569	19	1123379	0.09	0.13	0.52	0.15	0.08	0.08	0.10	0.21	0.65	1.06	0.50	0.03
rs4957256	5	40207040	0.02	0.15	0.88	0.09	0.08	0.31	<0.01	0.20	0.99	-0.05	0.53	0.93
rs503734	3	101304904	-0.12	0.2	0.55	-0.01	0.12	0.92	-0.20	0.26	0.44	-0.19	0.74	0.80
rs55946629	2	43624107	<0.01	0.16	0.98	/	/	/	-0.05	0.22	0.82	0.57	0.58	0.32
rs56062135	15	67163292	-0.14	0.12	0.25	0.07	0.07	0.33	-0.06	0.14	0.70	0.30	0.43	0.48
rs56116661	13	188683372	-0.33	0.18	0.07	-0.06	0.10	0.60	-0.22	0.28	0.45	0.19	0.64	0.77
rs56235845	5	177371039	-0.22	0.17	0.19	/	/	/	/	/	/	-0.10	0.62	0.88
rs5754100	22	21561877	-0.27	0.14	0.06	/	/	/	/	/	/	0.15	0.51	0.76
rs5763793	22	30130643	0.04	0.19	0.84	/	/	/	0.15	0.24	0.53	1.42	0.72	0.05
rs6017342	20	44436388	0.24	0.13	0.08	0.06	0.07	0.36	-0.06	0.15	0.68	-0.76	0.44	0.08
rs6062496	20	63697746	0.08	0.11	0.48	0.01	0.06	0.90	-0.31	0.13	0.02	0.12	0.37	0.75
rs6063502	20	50339058	0.09	0.21	0.66	<0.01	0.12	0.98	0.38	0.33	0.25	1.31	0.73	0.07
rs62126610	19	33257277	-0.19	0.14	0.17	<0.01	0.08	0.97	/	/	/	0.01	0.49	0.99
rs62183956	2	218181399	0.35	0.18	0.05	/	/	/	-0.45	0.23	0.04	-0.49	0.65	0.46
rs62324212	4	122639784	0.27	0.16	0.09	0.13	0.09	0.17	-0.04	0.22	0.86	0.65	0.58	0.26
rs62378712	5	159187037	-0.2	0.2	0.32	/	/	/	-0.16	0.28	0.57	/	/	/
rs62408218	6	90222139	-0.25	0.17	0.15	/	/	/	0.05	0.22	0.82	0.31	0.65	0.64
rs62482552	7	100924735	-0.24	0.2	0.22	-0.03	0.11	0.81	0.23	0.27	0.39	0.86	0.73	0.24
rs6457681	6	32805720	0.03	0.1	0.79	/	/	/	0.24	0.14	0.08	/	/	/
rs6579807	5	150907283	-0.09	0.16	0.55	0.08	0.09	0.42	0.17	0.20	0.38	0.13	0.62	0.83
rs6584282	10	99526738	0.06	0.09	0.48	0.05	0.05	0.35	0.15	0.11	0.17	-0.43	0.33	0.20
rs6674040	1	19875420	/	/	/	/	/	/				-0.26	0.45	0.57
rs6740847	2	181443625	-0.03	0.15	0.82	0.01	0.09	0.90	-0.04	0.21	0.84	0.71	0.56	0.20
rs6873866	5	96912106	-0.05	0.15	0.74	/	/	/	/	/	/	0.75	0.56	0.18
rs6933404	6	137638098	0.45	0.2	0.02	/	/	/	-0.34	0.24	0.16	0.34	0.72	0.64
rs714910	17	34290246	-0.02	0.16	0.92	/	/	/	-0.27	0.20	0.18	/	/	/
rs7190426	16	23844532	-0.07	0.19	0.72	-0.11	0.12	0.36	0.10	0.26	0.69	-0.14	0.73	0.85
rs7256518	19	10515699	0.03	0.19	0.87	0.02	0.11	0.83	0.08	0.23	0.72	0.35	0.67	0.60
rs72852162	2	145486323	/	/	/	0.07	0.12	0.54	/	/	/	-0.11	0.74	0.88
rs744166	17	42362183	-0.06	0.2	0.76	0.05	0.07	0.53	0.18	0.16	0.26	0.71	0.46	0.13
rs749910	16	50724938	0.02	0.12	0.84	-0.04	0.05	0.46	0.13	0.10	0.17	-0.01	0.30	0.97
rs7532133	1	160881744	-0.03	0.19	0.86	0.07	0.11	0.54	0.07	0.23	0.76	-0.24	0.69	0.73
rs7543680	1	22404776	/	/	/	<0.01	0.10	0.97				0.57	0.62	0.36
rs755374	5	159402286	-0.03	0.09	0.69	0.04	0.05	0.45	-0.03	0.11	0.78	0.01	0.32	0.96
rs7608697	2	60977506	-0.09	0.1	0.37	/	/	/	-0.33	0.12	0.01	-1.07	0.38	<0.01
rs76286777	2	24972708	0.07	0.17	0.69	0.07	0.10	0.47	-0.07	0.21	0.73	0.21	0.62	0.73
rs78771661	2	22129337	-0.3	0.18	0.1	0.06	0.08	0.49	-0.02	0.22	0.91	0.03	0.48	0.96
rs7918084	10	92669710	0.27	0.19	0.16	0.12	0.11	0.31	-0.12	0.25	0.62	2.07	0.72	<0.01
rs80262450	18	12818923	-0.18	0.14	0.2	-0.03	0.08	0.72	-0.10	0.18	0.57	-0.04	0.50	0.93
rs938650	8	128540294	-0.04	0.19	0.85	-0.13	0.11	0.24	-0.29	0.23	0.21	0.70	0.71	0.32
rs9934775	16	50349166	-0.03	0.16	0.86	/	/	/	0.10	0.27	0.72	0.88	0.62	0.16
All - Inverse variance weighted			-0.03	0.01	0.03	0.01	0.01	0.41	0.01	0.02	0.50	0.04	0.05	0.43
All - MR Egger			-0.02	0.03	0.66	-0.01	0.02	0.60	0.05	0.04	0.24	-0.22	0.13	0.08

AD: Alzheimer’s disease; ALS: amyotrophic lateral sclerosis; Chr: chromosome; IBD: inflammatory bowel disease; IVW: Inverse variance weighted; MS: multiple sclerosis; PD: Parkinson’s disease; SE: standard error; SNP: single-nucleotide polymorphism.

© 2022. Zeng R et al. Published online at http://www.aginganddisease.org/EN/10.14336/AD.2022.1209

SUPPLEMENTARY DATA

Supplementary Table 3. Mendelian randomization (MR) analysis for the causality ulcerative colitis (UC) and Crohn's disease (CD) on neurodegenerative disorders.

				Mendelian randomization				Heterogeneity	
**Exposure**	**Outcome**	**Method**	**SNPs**	**OR**	**LL**	**UL**	***P***	**Q**	**Q_*P***
CD	ALS	IVW	78	0.97	0.95	0.99	0.02	77.55	0.46
		MR Egger	78	1.02	0.96	1.09	0.52	75.01	0.51
		Weighted median	78	0.99	0.96	1.02	0.50		
		Weighted mode	78	1.00	0.94	1.05	0.87		
		MR PERSSO	78	0.97	0.95	0.99	0.02		
	AD	IVW	73	1.01	0.98	1.03	0.62	83.57	0.06
		MR Egger	73	0.98	0.93	1.04	0.56	83.38	0.05
		Weighted median	73	1.01	0.99	1.04	0.20		
		Weighted mode	73	1.02	0.99	1.05	0.30		
		MR PERSSO	73	1.01	0.98	1.03	0.62		
	PD	IVW	71	1.01	0.98	1.05	0.43	94.55	0.03
		MR Egger	71	1.09	1.00	1.18	0.05	89.96	0.05
		Weighted median	71	1.01	0.96	1.06	0.67		
		Weighted mode	71	1.00	0.94	1.07	0.97		
		MR PERSSO	71	1.00	0.94	1.07	0.97		
	MS	IVW	72	1.05	0.97	1.14	0.23	67.72	0.59
		MR Egger	72	1.05	0.84	1.32	0.66	67.72	0.55
		Weighted median	72	1.03	0.90	1.17	0.69		
		Weighted mode	72	1.00	0.84	1.21	0.96		
		MR PERSSO	72	1.05	0.97	1.14	0.23		
UC	ALS	IVW	52	1.01	0.98	1.03	0.68	40.34	0.86
		MR Egger	52	1.02	0.94	1.11	0.60	40.18	0.84
		Weighted median	52	1.00	0.96	1.04	0.94		
		Weighted mode	52	1.01	0.98	1.03	0.68		
		MR PERSSO	52	1.02	0.95	1.10	0.55		
	AD	IVW	41	1.01	0.99	1.03	0.28	38.98	0.52
		MR Egger	41	0.99	0.92	1.05	0.68	38.38	0.50
		Weighted median	41	1.01	0.98	1.04	0.48		
		Weighted mode	41	1.02	0.97	1.07	0.46		
		MR PERSSO	41	1.01	0.99	1.03	0.28		
	PD	IVW	49	0.99	0.96	1.03	0.78	51.30	0.35
		MR Egger	49	1.01	0.91	1.13	0.80	51.15	0.31
		Weighted median	49	1.01	0.96	1.06	0.78		
		Weighted mode	49	0.99	0.96	1.03	0.78		
		MR PERSSO	49	1.02	0.94	1.10	0.68		
	MS	IVW	50	0.88	0.78	0.99	0.03	57.52	0.19
		MR Egger	50	0.69	0.47	1.03	0.07	55.75	0.21
		Weighted median	50	0.97	0.82	1.14	0.70		
		Weighted mode	50	1.02	0.75	1.40	0.89		
		MR PERSSO	50	0.88	0.78	0.99	0.03		

AD: Alzheimer's disease; ALS: amyotrophic lateral sclerosis; CD: Crohn's disease; IVW: Inverse variance weighted; LL: lower limits of odds ratio; OR: Odds ratio; PD: Parkinson's disease; SNP: single-nucleotide polymorphism; UC: ulcerative colitis; MS: multiple sclerosis; UL: upper limits of odds ratio.

© 2022. Zeng R et al. Published online at http://www.aginganddisease.org/EN/10.14336/AD.2022.1209

SUPPLEMENTARY DATA

Supplementary Table 4. Assessment of pleiotropy.

		MR-Egger intercept			MR PRESSO global test	
**Exposure**	**Outcome**	**Intercept**	**SE**	***P***	**RSSobs**	***P***
IBD	ALS	-0.002	0.004	0.633	115.330	0.211
	AD	0.002	0.002	0.300	55.654	0.983
	PD	-0.006	0.006	0.329	116.912	0.033
	MS	0.036	0.016	0.024	127.537	0.043
UC	ALS	-0.003	0.006	0.690	42.179	0.865
	AD	0.004	0.005	0.442	40.847	0.488
	PD	-0.003	0.009	0.708	53.651	0.306
	MS	0.000	0.019	0.993	69.572	0.583
CD	ALS	-0.008	0.005	0.115	79.853	0.461
	AD	0.001	0.004	0.702	85.968	0.056
	PD	-0.014	0.007	0.065	97.520	0.033
	MS	<0.001	0.019	0.993	69.572	0.578

AD: Alzheimer’s disease; ALS: amyotrophic lateral sclerosis; Chr: chromosome; IBD: inflammatory bowel disease; IVW: Inverse variance weighted; MS: multiple sclerosis; PD: Parkinson’s disease; SE: standard error.

© 2022. Zeng R et al. Published online at http://www.aginganddisease.org/EN/10.14336/AD.2022.1209

SUPPLEMENTARY DATA

Supplementary Table 5. Mendelian randomization (MR) analysis for the causality of neurodegenerative disorders on inflammatory bowel disease (IBD), including ulcerative colitis (UC) and Crohn's disease (CD).

Exposure	Outcome	Method	SNPs	Mendelian randomization			
				OR	LL	UL	*P*
ALS	IBD	IVW	4	1.00	0.89	1.12	1.00
		MR Egger	4	0.92	0.70	1.20	0.59
		Weighted median	4	1.03	0.90	1.19	0.65
		Weighted mode	4	1.05	0.91	1.20	0.57
	UC	IVW	4	0.98	0.82	1.17	0.78
		MR Egger	4	0.77	0.54	1.10	0.29
		Weighted median	4	1.04	0.87	1.23	0.69
		Weighted mode	4	1.04	0.85	1.27	0.75
	CD	IVW	4	1.01	0.86	1.18	0.94
		MR Egger	4	1.04	0.67	1.62	0.87
		Weighted median	4	1.06	0.90	1.25	0.50
		Weighted mode	4	1.08	0.90	1.29	0.47
AD	IBD	IVW	42	0.99	0.94	1.05	0.84
		MR Egger	42	0.99	0.89	1.10	0.82
		Weighted median	42	0.99	0.92	1.08	0.84
		Weighted mode	42	0.99	0.91	1.08	0.78
	UC	IVW	44	1.02	0.94	1.11	0.68
		MR Egger	44	1.05	0.89	1.24	0.57
		Weighted median	44	1.04	0.94	1.15	0.48
		Weighted mode	44	1.05	0.94	1.18	0.37
	CD	IVW	44	1.02	0.91	1.14	0.75
		MR Egger	44	0.93	0.75	1.17	0.56
		Weighted median	44	1.05	0.94	1.18	0.37
		Weighted mode	44	0.94	0.81	1.09	0.42
PD	IBD	IVW	17	1.03	0.99	1.08	0.19
		MR Egger	17	1.07	0.96	1.19	0.25
		Weighted median	17	1.03	0.97	1.10	0.32
		Weighted mode	17	1.03	0.94	1.12	0.56
	UC	IVW	17	1.03	0.97	1.09	0.30
		MR Egger	17	1.02	0.89	1.18	0.74
		Weighted median	17	1.06	0.98	1.14	0.15
		Weighted mode	17	1.09	0.97	1.22	0.16
	CD	IVW	17	1.04	0.98	1.10	0.24
		MR Egger	17	1.08	0.94	1.24	0.31
		Weighted median	17	1.03	0.95	1.12	0.48
		Weighted mode	17	1.01	0.88	1.15	0.89
MS	IBD	Wald ratio	1	0.94	0.86	1.03	0.18
	UC	Wald ratio	1	1.09	0.97	1.22	0.15
	CD	Wald ratio	1	1.03	0.92	1.16	0.56

IBD: inflammatory bowel disease; AD: Alzheimer's disease; ALS: amyotrophic lateral sclerosis; CD: Crohn's disease; IVW: Inverse variance weighted; LL: lower limits of odds ratio; MS: multiple sclerosis; OR: Odds ratio; PD: Parkinson's disease; SNP: single-nucleotide polymorphism; UC: ulcerative colitis; UL: upper limits of odds ratio.

© 2022. Zeng R et al. Published online at http://www.aginganddisease.org/EN/10.14336/AD.2022.1209

SUPPLEMENTARY DATA

Supplementary Table 6. Risk loci associated with inflammatory bowel disease (IBD) conditional on amyotrophic lateral sclerosis (ALS).

SNP	Allele1	Allele2	*P*	Chr	Position	GWAS FDR	Gene
rs6571361	A	G	2.54E-07	14	31183168	3.48E-02	*SCFD1*
rs7154847	A	G	4.46E-07	14	31059969	2.70E-02	*G2E3*

FDR: false discovery rate; Chr: chromosome; SNP: Single nucleotide polymorphism.

Supplementary Table 7. Variant status and the risk of inflammatory bowel disease (IBD) and amyotrophic lateral sclerosis (ALS).

Outcome	Factor	Adjusted Odds Ratio*	*P*
**rs6571361**			
IBD	Noncarrier	1 (Reference)	
	Heterozygous carrier	0.99 (0.94-1.04)	0.73
	Homozygous carrier	0.96 (0.87-1.05)	0.33
ALS	Noncarrier	1 (Reference)	
	Heterozygous carrier	1.10 (0.90-1.35)	0.36
	Homozygous carrier	1.42 (1.16-1.69)	**0.03**
**rs7154847**			
IBD	Noncarrier	1 (Reference)	
	Heterozygous carrier	0.99 (0.94-1.05)	0.78
	Homozygous carrier	0.97 (0.88-1.06)	0.52
ALS	Noncarrier	1 (Reference)	
	Heterozygous carrier	1.10 (0.90-1.35)	0.36
	Homozygous carrier	1.48 (1.09-1.77)	**0.01**

ALS: amyotrophic lateral sclerosis; IBD: inflammatory bowel disease.
*Adjusted for age, sex, Townsend deprivation index, ethnicity, alcohol consumption, smoking status, metabolic equivalent of task, and body mass index.

© 2022. Zeng R et al. Published online at http://www.aginganddisease.org/EN/10.14336/AD.2022.1209

SUPPLEMENTARY DATA

Supplementary Table 8. Functional enrichment of shared risk loci.

Category	Pathway (ID)	*P* value	Adjusted *P* value
Biological Process	regulation of ER to Golgi vesicle-mediated transport (GO:0060628)	0.0013	0.0149
	negative regulation of autophagosome assembly (GO:1902902)	0.0019	0.0149
	regulation of establishment of protein localization (GO:0070201)	0.0022	0.0149
	negative regulation of macroautophagy (GO:0016242)	0.0038	0.0149
	regulation of protein transport (GO:0051223)	0.0041	0.0149
	regulation of autophagosome assembly (GO:2000785)	0.0052	0.0149
	negative regulation of organelle assembly (GO:1902116)	0.0052	0.0149
	regulation of transport (GO:0051049)	0.0055	0.0149
	regulation of intracellular transport (GO:0032386)	0.0058	0.0149
	COPII vesicle coating (GO:0048208)	0.0094	0.0180
	vesicle coating (GO:0006901)	0.0094	0.0180
	vesicle targeting, rough ER to cis-Golgi (GO:0048207)	0.0094	0.0180
	COPII-coated vesicle budding (GO:0090114)	0.0104	0.0185
	retrograde vesicle-mediated transport, Golgi to endoplasmic reticulum (GO:0006890)	0.0126	0.0191
	retrograde transport, endosome to Golgi (GO:0042147)	0.0131	0.0191
	post-Golgi vesicle-mediated transport (GO:0006892)	0.0132	0.0191
	regulation of vesicle-mediated transport (GO:0060627)	0.0143	0.0193
	cytosolic transport (GO:0016482)	0.0173	0.0221
	endoplasmic reticulum to Golgi vesicle-mediated transport (GO:0006888)	0.0274	0.0332
	protein-containing complex assembly (GO:0065003)	0.0395	0.0454
Cellular Component	Golgi-associated vesicle (GO:0005798)	0.0070	0.0298
	cis-Golgi network (GO:0005801)	0.0085	0.0298
Molecular Function	syntaxin binding (GO:0019905)	0.0092	0.0092

GO, Gene Ontology.

© 2022. Zeng R et al. Published online at http://www.aginganddisease.org/EN/10.14336/AD.2022.1209
